# Focal atrial tachycardia from the superior vena cava following atrial fibrillation cryoablation: a case report

**DOI:** 10.3389/fcvm.2026.1888774

**Published:** 2026-07-02

**Authors:** Radu-Gabriel Vatasescu, Gabriela-Elena Marascu, Viviana Alexandra Gondos, Alexandru Ioan Deaconu

**Affiliations:** 1 Faculty of Medicine, Carol Davila University of Medicine and Pharmacy, Bucharest, Romania; 2 Department of Cardiology, Clinical Emergency Hospital of Bucharest, Bucharest, Romania; 3 Department of Medical Electronics and Informatics, Polytechnic University of Bucharest, Bucharest, Romania

**Keywords:** atrial fibrillation, atrial tachycardia, cryoablation, electrophysiological study, superior vena cava

## Abstract

A 58-year-old woman with recurrent paroxysmal atrial fibrillation and a history of cryoablation 3 years prior underwent an electrophysiological study for an irregular narrow complex tachyarrhythmia. The electrocardiogram showed positive P’ waves in all leads except aVR, with the P’ waves separated by large isoelectric intervals, indicating focal atrial tachycardia rather than atypical atrial flutter. High-resolution 3D mapping revealed a focal atrial tachycardia originating from the postero-septal part of the superior vena cava (in close proximity to the right superior pulmonary vein). Three applications at the superior vena cava level terminated the tachycardia and restored sinus rhythm. There were no recurrences. This case underscores the need for high-resolution electrophysiological mapping to identify the arrhythmia origin, especially in patients with prior procedures.

## 1 Introduction

Focal or macroreentrant atrial tachycardia (AT) may arise as a result of prior catheter ablation for atrial fibrillation (AF) or after cardiac surgery (1). The mechanisms underlying AT are characterized through high-density electroanatomic mapping and entrainment responses, which help pinpoint the sites of origin, which are often marked by low-voltage electrograms, fragmentation, and prolonged duration (1). The arrhythmogenic substrate most commonly involves the reconnection of pulmonary veins (PVs). However, the superior vena cava (SVC) has been identified occasionally as a key source of atrial ectopy that can trigger AT. We report a case of focal AT originating from the SVC following prior cryoablation for paroxysmal AF.

## 2 Case presentation

A 58-year-old woman with a history of recurrent paroxysmal AF and typical atrial flutter presented for a redo radiofrequency catheter ablation procedure 3 years after an index cryoballoon pulmonary vein isolation. Her past medical history included arterial hypertension, type 2 diabetes mellitus treated with oral antidiabetic agents, and dyslipidemia. Her CHA₂DS₂-VA score was 2, and she was receiving apixaban 5 mg twice daily. She reported irregular palpitations and fatigue. A 12-lead ECG showed an irregular supraventricular tachyarrhythmia with a rate of 90 bpm, raising a differential diagnosis between focal atrial tachycardia and atypical atrial flutter (Figure 1). Transthoracic echocardiography showed a left ventricle with preserved systolic function, no hemodynamically significant valvular disease, and a mildly dilated left atrium (LA). All baseline assessments, including laboratory investigations and physical examination, were normal.

**Figure 1:**
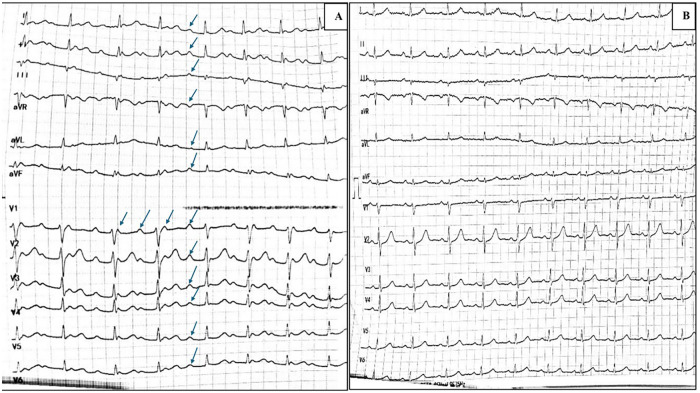
Electrocardiograms: narrow complex tachyarrhythmia at 90 bpm with variable atrioventricular block and positive P wave in all leads, except aVR (indicated by the blue arrows) **(A)**; and sinus rhythm, with 70 bpm **(B)**.

At the beginning of the electrophysiological study (EPS), the patient was in sinus rhythm (SR). 3D electroanatomical mapping of the LA was performed (CARTO 3, Biosense Webster, Irvine, CA, United States), revealing reconnection of the right inferior pulmonary vein (Figure 2A). Radiofrequency applications were delivered at this site until a bidirectional block between the pulmonary vein and the left atrium was achieved. A cavotricuspid isthmus ablation line was also created, and it was then confirmed that all four pulmonary veins and the isthmus remained isolated. However, spontaneous AT appeared at the end of the right atrium (RA)/LA remapping. The tachycardia remained ongoing, with high-resolution mapping showing activation signals localized to the earliest activation site on the postero-septal wall of the SVC (Figures 3A,B). Three focal radiofrequency applications were delivered at this site, resulting in termination of the tachycardia and restoration of a stable SR (Figures 3C,D). Subsequently, an ultrahigh-resolution electroanatomical map was created using the Optrell catheter, allowing detailed characterization of local electrograms and activation patterns (Figures 3E,F). No additional lesions were delivered to achieve complete SVC isolation. Given the proximity of the superior vena cava to the right phrenic nerve, phrenic nerve function was monitored via high-output pacing during ablation. No reduction in diaphragmatic capture was observed, and no phrenic nerve injury occurred. Following focal radiofrequency ablation, programmed atrial stimulation was performed to assess arrhythmia inducibility. No sustained atrial tachycardia could be induced, confirming the procedural endpoint.

**Figure 2:**
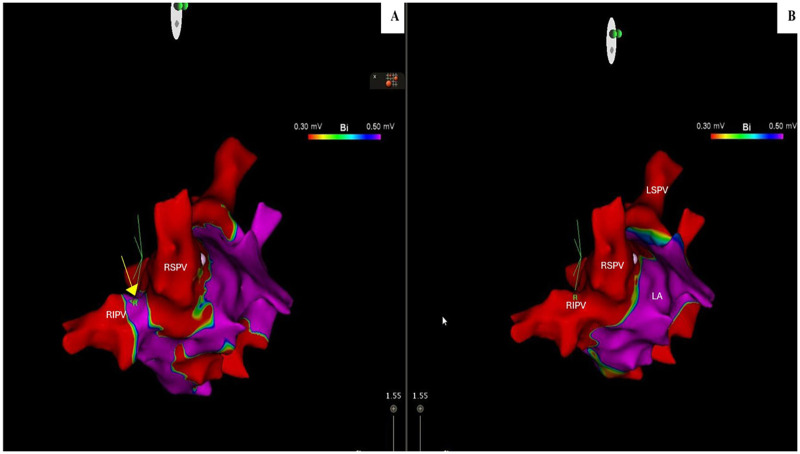
3D electroanatomic mapping before **(A)** and after **(B)** radiofrequency isolation of the right inferior pulmonary vein. The right inferior pulmonary vein reconnection is indicated by the yellow arrow. LIPV, left inferior pulmonary vein; RSPV, right superior pulmonary vein; LA, left atrium; LSPV, left superior pulmonary vein.

**Figure 3:**
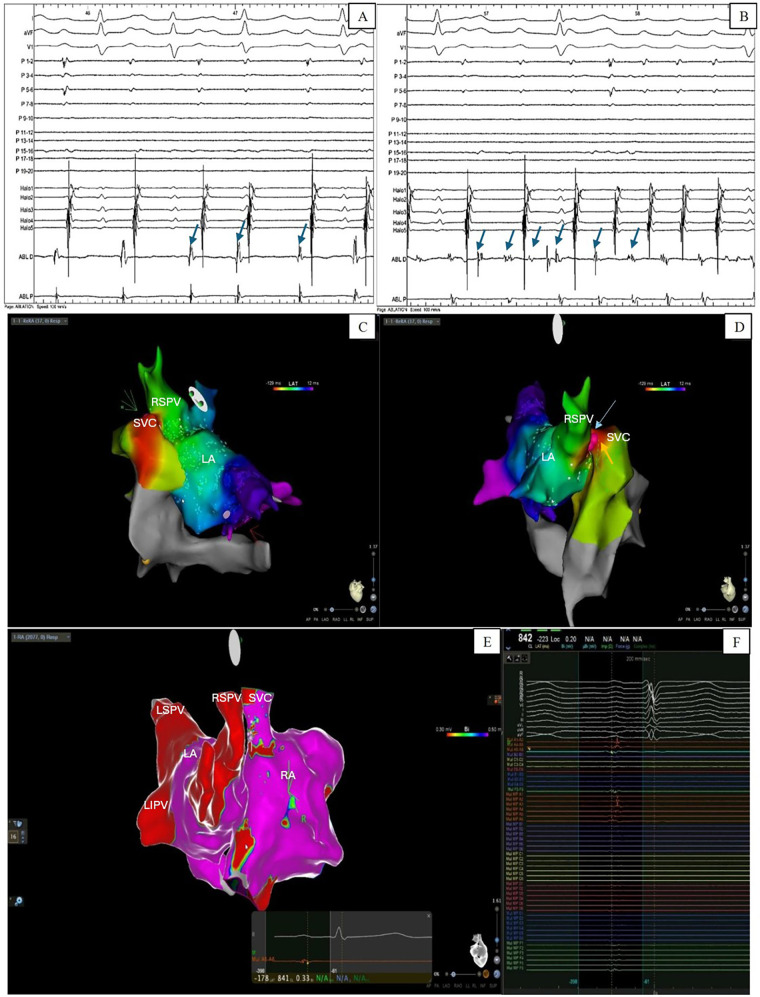
Electrophysiological study with 3D electroanatomical mapping: **(A)** local signals at the level of the superior wall of the RA, near the SVC (blue arrows); **(B)** fragmented electrograms at the level of the posterior wall of the SVC (blue arrows); **(C**,**D)** atrial tachycardia localization: 3D electroanatomical activation mapping demonstrating the earliest site of activation within the SVC (red area, indicated by the orange arrow) and location of radiofrequency applications (pink points, indicated by the blue arrow) that led to the termination of atrial tachycardia; **(E)** 3D ultrahigh-density electroanatomic mapping with Optrell multipolar catheter revealing a relatively large low-voltage area at the level of the superior and posterior wall of the RA–superior vena cava junction that mirrors the contact area with right superior pulmonary vein; and **(F)** fragmented electrograms in the same area. RIPV, right inferior pulmonary vein; RSPV, right superior pulmonary vein; LA, left atrium; LSPV, left superior pulmonary vein; SVC, superior vena cava.

During 1 year of follow-up, the patient remained asymptomatic. She continued anticoagulation with apixaban and did not require antiarrhythmic medication after the procedure. Serial outpatient evaluations, 12-lead electrocardiograms, and repeated 24-h Holter ECG recordings consistently demonstrated a stable sinus rhythm with no evidence of arrhythmic recurrence. A summary of the clinical course of the patient is presented in Figure 4.

**Figure 4:**
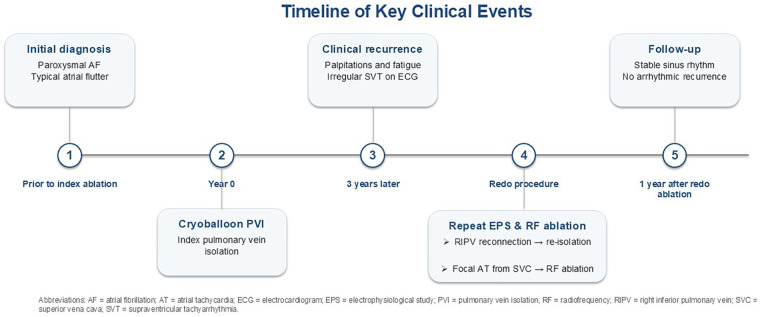
Summary of the clinical course of the patient, including index cryoballoon ablation, recurrence, repeat electrophysiological study, and follow-up.

## 3 Discussion

After pulmonary vein isolation, patients may experience organized ATs due to incomplete ablation lines, atrial scar tissue, or ectopic foci outside the pulmonary veins. The incidence of AT after AF ablation ranges from less than 5% to 40% and depends on the ablation technique (2). Focal AT, often occurring after paroxysmal AF ablation and due to reconnected pulmonary veins, accounts for up to 80% of cases, while macroreentrant AT occurs after extensive left atrial ablation (2, 3). Lyan et al. reported that AT occurred fairly often after cryoballoon ablation, with an incidence of 11.3%, and macroreentry was the most common underlying mechanism (4). Chang et al. found a higher incidence of left atrial flutter after cryoablation (54.5%) compared with the radiofrequency strategy (12.5%), likely due to more extensive low-voltage areas in the left atrium resulting from cryoablation (5).

Recently, Peng et al. showed that 15.9% of patients developed right-sided AT following initial AF ablation (6). Among these, typical atrial flutter was the most frequent form (62.9%), while focal AT originating from the SVC ostium was the most common non-cavotricuspid isthmus-dependent subtype (17.1%) (6). The differential diagnosis for our patient included atypical atrial flutter, particularly in the setting of prior AF ablation. Although surface ECG findings suggested a high right atrial or SVC origin, ECG interpretation alone was insufficient to reliably distinguish focal AT from a macroreentrant arrhythmia. The mechanism was clarified by high-density activation mapping, which showed the earliest activation site within the SVC followed by centrifugal spread to the atria, without evidence of a macroreentrant activation sequence.

The SVC and pulmonary veins share several similarities, including atrial myocardial sleeves that extend into their structures. They are also spatially connected, as the SVC is adjacent, anterior, and superior to the right superior pulmonary vein (7). SVC-related AT is typically focal, with the earliest activation near the SVC–right atrial junction, typically due to residual or unmasked arrhythmogenic activity within the SVC myocardial sleeves. It has been reported to have a characteristic P-wave morphology, representing a highly positive P-wave in leads I, II, III, aVF, an isoelectric P-wave in lead aVL, a biphasic (positive/negative) P-wave in lead V1, and a positive P-wave in leads V2–V6 (8). An electrophysiology study with 3D electroanatomical mapping is essential for diagnosing and localizing this arrhythmia. It shows centrifugal activation from a single point and confirms effective target sites when the tachycardia accelerates during ablation (2). In our case, reconnection of the right inferior pulmonary vein was noted, and the origin of the AT was identified on the posterior wall of the SVC. This was confirmed by tachycardia acceleration during the EPS and its termination following radiofrequency applications at that site (Figure 3). Due to the presence of a low-voltage area with fragmented signals at the RA–SVC junction, as well as extremely fragmented and wide local electrograms at the site of AT origin, we hypothesize that prior right superior pulmonary vein cryoablation induced fibrosis and conduction changes that extended to the posterior SVC, potentially explaining the localized reentry or focal atrial tachycardia. Although prior cryoballoon ablation may have contributed to local remodeling and conduction abnormalities, a causal relationship cannot be established. Alternative explanations include a pre-existing arrhythmogenic focus within the SVC or progressive atrial cardiomyopathy resulting in substrate formation independent of the index ablation procedure.

## 4 Conclusion

AT arising from the SVC is a recognized but uncommon arrhythmia that can occur after pulmonary vein isolation for AF. An electrophysiological study with high-resolution electroanatomic mapping is necessary to confirm the SVC origin and differentiate focal from macroreentrant mechanisms.

## Data Availability

The original contributions presented in the study are included in the article; further inquiries can be directed to the corresponding author.
